# Long-Term Experience with Balloon Dilation for Short Bulbar and Membranous Urethral Strictures: Establishing a Baseline in the Active Drug Treatment Era

**DOI:** 10.3390/jcm11113095

**Published:** 2022-05-30

**Authors:** Lauren A. Beeder, Grayden S. Cook, Samantha W. Nealon, Shervin Badkhshan, Sarah C. Sanders, Dylan P. Perito, Steven J. Hudak, Allen F. Morey

**Affiliations:** Department of Urology, University of Texas Southwestern Medical Center, Dallas, TX 75390, USA; lauren.beeder@phhs.org (L.A.B.); grayden.cook@utsouthwestern.edu (G.S.C.); samantha.wardnealon@utsouthwestern.edu (S.W.N.); shervin.badkhshan@utsouthwestern.edu (S.B.); sarah.sanders@utsouthwestern.edu (S.C.S.); dylan.perito@utexas.edu (D.P.P.); steven.hudak@utsouthwestern.edu (S.J.H.)

**Keywords:** balloon dilation, urethral stricture, success rate

## Abstract

Transurethral balloon dilation (BD) is a minimally invasive treatment for urethral stricture disease (USD) performed primarily or as a recurrence salvage maneuver. With the introduction of drug-coated balloons, we sought to characterize patient outcomes using non-medicated balloons. A retrospective review identified patients who underwent BD from 2007 to 2021. Patient and stricture characteristics were collected. All dilations employed the 24Fr UroMax^TM^ system. Clinical failure was defined by patient-reported lower urinary tract symptom recurrence or need for further stricture management. Ninety-one patients underwent BD with follow-up median (IQR) 12 (3–40) months. Most (75/91, 82%) had prior treatment for USD (endoscopic 50/91 (55%), 51/91 (56%) urethroplasty) before BD. Recurrence rates did not significantly differ between treatment-naïve and salvage patients (44% vs. 52% (*p* = 0.55)). Median (IQR) time to failure was 6 (3–13) months. The most common complications were urinary tract infection (8%) and post-operative urinary retention requiring catheterization (3%). Radiation history was noted in 33/91 (36%) with 45% recurrence. Patients without previous radiation had a similar recurrence rate of 52% (*p* = 0.88). Balloon dilation had minimal complications and overall, 50% recurrence rate, consistent regardless of stricture characteristics, radiation history, or prior treatments. These results represent an important clinical benchmark for comparing outcomes using drug-coated balloons.

## 1. Introduction

Urethral stricture disease is a relatively common condition that affects 0.6% of men in the United States [1,2]. Treatment options for strictures largely depend on the type, length, and location of stricture, as well as patient characteristics and surgeon expertise. In general, options include coaxial dilation, balloon dilation (BD), direct vision internal urethrotomy (DVIU), and urethroplasty. The most common treatments offered among urologists in the United States are dilation and DVIU [3,4].

Urethroplasty is considered the gold standard for definitive treatment of urethral stricture disease. This is due to the high rate of stricture recurrence with endoscopic management and the increased complexity of recurrent strictures following repeated endoscopic treatment [5]. Current AUA guidelines state that patients should be offered urethroplasty following failure of initial endoscopic procedures [6]. However, urethroplasty requires general anesthesia, an experienced surgeon, and is associated with a longer recovery time and indwelling catheter duration. Alternatively, BD is a safe, well-tolerated procedure, which can be performed in the outpatient clinic. It is a procedure with which all practicing urologists are familiar and comfortable.

The recent and ongoing ROBUST trials have introduced a paclitaxel-coated balloon system, now FDA-approved in the USA, and have demonstrated their safety and efficacy for the treatment of urethral stricture disease [7,8]. In light of the introduction of this novel balloon system, we sought to establish a fundamental clinical baseline by evaluating our experience with “traditional” BD. Herein, our objective is to report our recurrence rates and delineate risk factors for BD failure.

## 2. Materials and Methods

We retrospectively reviewed the institutional database at our tertiary referral center and identified patients who underwent BD for urethral stricture disease between 2007 and 2021. Patient characteristics were collected, including age, race, medical comorbidities, smoking status, history of radiation therapy, surgical history, history of prior procedures, and surgeries for urethral stricture disease. Stricture characteristics were also compiled—location, length, etiology (trauma vs. radiation vs. idiopathic), and number of strictures.

All BDs were performed using a 16Fr cystoscope under direct vision with an 8 cm, 24F UroMax Ultra™ balloon dilator (Boston Scientific Corp, Marlborough, MA, USA) over an Amplatz Super Stiff ™ Guidewire (Boston Scientific Corp.) Balloons were inflated to 20 atmospheres of pressure for 4 minutes. A 20-French-Council-tip catheter was then advanced over the wire and left in place for 24 h. We compared successful BDs to failures. Procedure failure was defined by recurrence of lower urinary tract symptoms (LUTS) or need for further stricture treatment. When available, we reviewed procedure reports from post-operative cystoscopies and compared objective findings with patient reports of symptoms.

## 3. Results

Our patient cohort consisted of 91 patients who underwent BD for urethral stricture disease with median (IQR) follow-up time of 12 months (3–40). The mean age at time of BD was 61 years. History of prostate cancer was identified in 37/91 (41%) of patients, and 33/91 (36%) had a history of radiation. Radiation modalities included external beam radiation therapy (EBRT) (21/33, 64%), brachytherapy (BT) (10/33, 30%), or a combination of the two (2/33, 6%). Other relevant patient demographic and clinical information is listed in Table 1.

Prior intervention for urethral stricture disease was seen in 75/91 (82%) of patients, with 55% (50/91) having undergone prior endoscopic treatment and 56% (51/91) prior urethroplasty. Among patients with prior stricture management, 38% (35/91) had at least two prior endoscopic treatments, and 16% (15/91) had two or more prior urethroplasties. The majority of strictures were located in the bulbar urethra (75/91 (82%)) and membranous urethra (16/91 (18%)).

The overall recurrence rate of BD was 46/91 (50%). In the patients who reported recurrence of their LUTS, median (IQR) time to failure was 6 (3–13) months. No comparisons between demographic or clinical factors achieved statistical significance for those with successful treatment vs. those who failed (Table 1). No stricture characteristics were predictive of BD failure. Furthermore, there was no difference in recurrence rates of BD in treatment-naïve vs. salvage patients. Moreover, 12 patients out of 45 (27%) had follow-up cystoscopy vs. 31/46 (67%) of those with dilation failure (*p* = 0.0001). Of those who had follow-up cystoscopy, those with treatment success were much more likely to have a cystoscopically patent urethra of >16Fr compared to those with treatment failure: 10/12 (83%) and 9/31 (29%), respectively (*p* = 0.0001).

### 3.1. Complications

The most common complication within 30 days after BD was the development of urinary tract infection (UTI), with 7/91 (8%) patients requiring antibiotic treatment. Post-operative gross hematuria was identified in 2/91 (2%) of patients, while no patients required intraoperative or postoperative blood transfusions. Postoperative urinary retention requiring emergency room visit occurred for 3/91 (3%) of patients. No patients required readmission for a urologic problem within 30 days postoperatively.

### 3.2. Irradiated vs. Non-Irradiated Patients

Patients with a history of radiation had significantly higher mean age at time of BD (71 years, *p* = <0.0001), lower incidence of prior trauma (*p* = 0.04), and a higher incidence of hypertension (*p* = 0.04). Follow-up cystoscopy was performed in 16/33 (48%) of the irradiated group vs. 27/58 (47%) in the non-irradiated group. There was no difference in rates of urethral patency (>16Fr) on post-operative cystoscopy between irradiated and non-irradiated patients (*p* = 0.86). Table 2 displays the relationships between follow-up cystoscopic findings and the presence of LUTS. There was no correlation between report of symptoms and urethral lumen size on cystoscopy.

Recurrence was seen in 15/33 (45%) patients who underwent radiation compared to 28/58 (48%) who did not receive radiation (*p* = 0.57). Among irradiated patients who failed, median (IQR) time to failure was 7 (3–14) months vs. a median (IQR) of 5 months (3–13) in the non-irradiated cohort. Patients in the radiation group were much more likely to have suprapubic tube placement (*p* = 0.01) vs. patients in the non-irradiated group who underwent significantly more urethroplasty (*p* = 0.003).

### 3.3. Primary vs. Salvage Patients

For 16/91 (18%) patients, BD was the first documented treatment for their urethral stricture disease. Patients with and without prior treatment did not differ significantly in terms of demographic or clinical factors. Furthermore, stricture characteristics including location and length were not significantly different between groups.

Follow-up cystoscopy was performed in 7/16 (44%) of the treatment-naive patients vs. 36/75 (48%) of patients with prior treatment. This revealed no significant differences between the two groups across any of the cystoscopic/clinical combinations of >16Fr urethral patency and the presence of LUTS. Balloon dilation failed in 44% (7/16) of the treatment-naive cohort compared to 52% (39/75) in those with history of other treatment (*p* = 0.55). The median (IQR) time to failure in treatment-naive patients was 4 months (1–36) compared to patients with prior treatment with a median (IQR) of 5 months (3–11) months (*p* = 0.02).

## 4. Discussion

**Figure 1:**
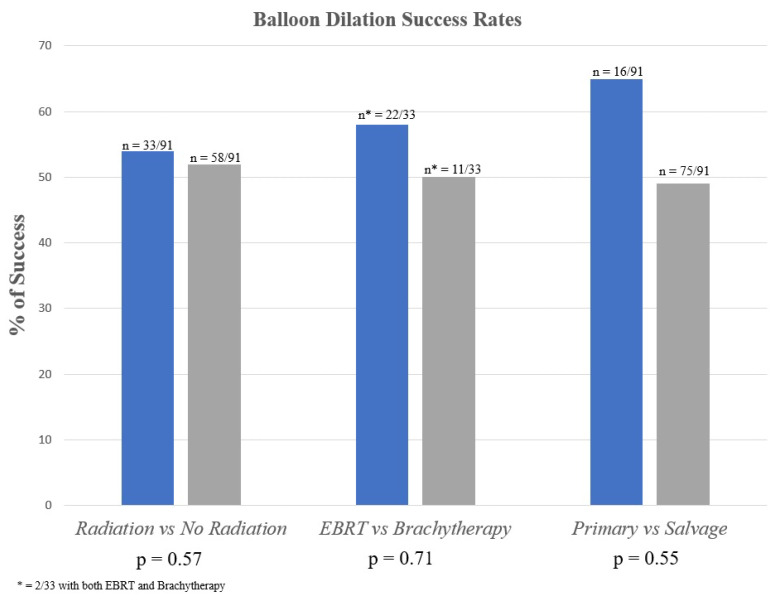
Balloon Dilation Success Rates.

Drug-coated balloons will be more costly than traditional balloon dilators and thus should be expected to perform with a level of clinical efficacy justifying that additional expense. We sought to review our extensive experience with traditional BD over the past decade and provide a clinical baseline for the purpose of outcome comparison and for future drug-coated BD studies. Our data analysis was largely consistent with prior studies which have demonstrated about a 50% recurrence rate, with a median time to recurrence of 5 (2–11) months. Patients with history of prior radiation or endoscopic treatment/urethroplasty did not fare significantly worse than patients without radiation or prior treatment (Figure 1). Additionally, the rate of significant complications was negligible. As such, BD is an option that should be offered to patients who are unable or unwilling to undergo urethroplasty or as a short-term symptom-relief measure before more definitive treatment can be pursued.

As a tertiary care center with a large referral pattern, many of the patients who receive care at our institution for urethral stricture disease (USD) have first undergone treatment at outside centers and are referred for recurrence of USD. Most patients in our cohort had at least one prior endoscopic procedure or urethroplasty, and many had multiple prior procedures. Earlier data from this institution have shown that BD performs poorly as a salvage procedure for urethral strictures that recur following urethroplasty [9]. In this cohort, there was no significant difference in recurrence rates between primary and salvage cases.

Balloon dilation appears to be a safe procedure. In our cohort, very few patients experienced complications as a result of BD. The most common complication was UTI, and a small percentage of patients experienced urinary retention requiring catheter replacement. The low complication rate is likely related to the mechanism of BD, which applies radial force to strictures under direct vision and is generally less traumatic with a lower bleeding risk and infection rate compared to blind sequential dilation or DVIU [10]. One study directly comparing BD to DVIU revealed shorter operative times and lower complication rates with BD [11].

Urethral strictures secondary to a history of radiation are relatively common and can be particularly difficult to treat due to compromised vascular supply, tissue necrosis, altered tissue planes, and poor wound healing. Strictures occur in 2% of EBRT patients, 4–32% of BT patients, and 11% of patients treated with combination therapy [12,13,14]. For short, radiation-induced strictures, anastomotic urethroplasty has been shown to have the highest rates of success, approaching 95%. Comparatively, endoscopic treatments have been shown to have higher rates of stricture recurrence, such as DVIU recurrence rates of nearly 50% [15]. A significant portion of the patients in our cohort (38%) had a history of radiation, but there was no difference in recurrence rates between irradiated and non-irradiated patients. These findings suggest that BD remains an option for the treatment of urethral strictures in radiation-induced stricture disease.

Lastly, outcomes in this study were largely based on patient reports of symptoms. Approximately half of patients underwent post-operative cystoscopic evaluation. We defined treatment failure as the recurrence of symptoms or the need for additional surgical or endoscopic intervention. There did not appear to be a correlation between cystoscopic findings of urethral narrowing (<16Fr) and patient symptoms. This finding confirms existing data from multiple studies (including a prospective, multi-institutional study), which have demonstrated that the presence of urethral stricture on cystoscopic evaluation was a poor predictor of patient symptoms. These findings support the use of patient-reported symptoms, rather than objective measures such as cystoscopy or urine flow data, as a guide for repeat intervention [16,17].

### Limitations

Our study had several limitations including its retrospective nature. We recognize that the lack of standardized patient reporting measures for post-operative symptomatology as well as the lack of anatomic evaluation of stricture recurrence in approximately half of patients also represent significant limitations. Another limitation is the potential for self-selection bias since patients who followed up may have been more likely to experience symptoms of recurrence post-operatively. Finally, this was a single-center study, and the findings may not be generalizable.

## 5. Conclusions

The endoscopic balloon dilation of short membranous and bulbar strictures is a safe and technically straightforward procedure with a recurrence rate of approximately 50%, independent of prior radiation or surgical treatment history.

## Figures and Tables

**Table 1:** Clinical/Stricture Characteristics of Success vs. Failure Patients.

Clinical Factor	Successful Patients *n* = 45/91 (49%)	Failed Patients *n* = 46/91 (51%)	*p*-Value
*CAD*	14	7	0.07
*DM*	8	12	0.34
*HTN*	22	29	0.17
*HLD*	23	19	0.35
*COPD*	2	0	0.24
*CHF*	2	2	1
*Smoking History*	23	19	0.35
*Malignancy History*	18	19	0.99
*History of Trauma*	5	7	0.26
*Age at Surgery Mean (SD)*	60 (17)	62 (12)	0.26
*BMI Mean (SD)*	30 (6)	31 (8)	0.15
**Stricture Location (*n*)**	-	-	-
*Bulbar*	36/45 (80%)	39 (85%)	0.55
*Membranous*	9/45 (20%)	7 (15%)	
**Prior Management (*n*)**	-	-	-
Prior Endoscopic Management *	23/45 (51%)	27/46 (59%)	0.47
*Prior Urethroplasty*	22/45 (49%)	29/46 (63%)	0.17
*Timing from Latest Treatment* *to Balloon: Median Months*	9.5 (4–29)	6 (4–22)	0.27

* Prior Endoscopic Management = Catheter/Balloon Dilation, Direct Visual Internal Urethrotomy; Anterior = Bulbar and Penoscrotal; Posterior = Membranous, Prostatic and Bladder Neck Coronary artery disease (CAD), diabetes mellitus (DM), hypertension (HTN), hyperlipidemia (HLD), chronic obstructive pulmonary disease (COPD), congestive heart failure (CHF), body mass index (BMI).

**Table 2:** Balloon Outcomes in Irradiated vs. Non-Irradiated Patients.

	Irradiated Patients *n* = 33/91 (36%)	Non-Irradiated Patients *n* = 58/91 (64%)	*p*-Value
**Follow-up Cystoscopy Findings**	*n* = 16/33 (48%)	*n* = 27/58 (47%)	-
*>16Fr*	8	11	0.86
*<16Fr*	8	16	
**Symptom/Cystoscopic** **Concordance**	-	-	-
*No Sx and >16Fr*	3	7	0.7
*No Sx and <16Fr*	0	3	0.28
*Sx and >16Fr*	5	4	0.26
*Sx and <16Fr*	8	13	0.91
**Failed Patients**	*n* = 15/33 (45%)	*n* = 28/58 (48%)	0.57
*Median (IQR) Time to Failure in Months*	7 (3–13)	5 (3–13)	0.33
**Management Post-Balloon** **Failure**	*n* = 15/33 (45%)	*n* = 27/58 (47%)	-
*Catheter Dilation*	0	4	0.28
*DVIU*	2	1	0.25
*Repeat Balloon Dilation*	5	3	0.09
*Suprapubic Tube*	6	2	0.01 *
*Urethroplasty*	1	16	0.003 *
*Perineal Urethrostomy*	0	0	1
*Urinary Diversion*	1	1	1

DVIU = Direct Vision Internal Urethrotomy; Sx = Symptom; 16Fr = 16 French diameter urethral lumen. * indicate statistically significant *p*-values.

## Data Availability

Not applicable.
